# The Teachings of Adversity

**Published:** 1895-11

**Authors:** 


### The Teachings of Adversity.

The Bitter One—"I tell you, a man changes his mind about his friends and enemies."

"How so, old man?"

"His enemies stop hitting him when he's down, but it's then that his friends begin."

